# The Level of Aggressiveness During Karate Practice of Inmates in Correctional Settings

**DOI:** 10.3389/fpsyg.2020.567668

**Published:** 2020-10-02

**Authors:** Jérôme Frigout, Olivier Degrenne, Arnaud Delafontaine

**Affiliations:** ^1^I3SP Laboratory, Department of Sports Science and Physical Education, Université de Paris Descartes, Paris, France; ^2^LIRTES, Université Paris-Est Créteil, Créteil, France; ^3^CIAMS, Université Paris-Sud, Université Paris-Saclay, Orsay, France; ^4^CIAMS, Université d’Orléans, Orléans, France

**Keywords:** karate, combat sports, prison, aggressiveness, behavior

## Abstract

Karate is known to enhance cognitive functioning, emotional well-being, and self-regulation and to contribute to an overall behavior rehabilitation process. However, few data are available on the impact of practicing karate in adult prison inmates. The main objective of this research was to evaluate aggressive behavior, comparing prison inmates and club practitioners during karate practice. The level of aggressiveness was rated by observers during defined elements and training situations in karate classes held in France. Data were collected during 77 observations of 75 prison inmates (55 male and 20 female) in a prison setting, and 188 observations of 117 club practitioners (80 male and 37 female) in a club setting over a period of 26 months. Licit aggressiveness was graded by observers during launched actions, *kiais*, and bows, and the practice level (belts) was also considered. Interrater reliability of the observational measure was highly acceptable (Cohen κ = 1). Comparisons between female and male prison inmates and club practitioners were made using the non-parametric Mann–Whitney *U*-test for independent samples. The results revealed that a higher level of aggressiveness was observed in both male and female club practitioners during launched actions and *kiais* than in prison inmate practitioners (*p* < 0.001, small effect size). However, prison inmates (of both genders) showed a higher level of aggressiveness during bows (*p* < 0.001, medium effect size). While the analyses showed no significant differences between genders, the level of karate practice was associated with distinct changes. Significant differences in observed aggressiveness were present only in beginners and in those with a low level of karate practice, whereas no differences in aggressiveness between prison or club practitioners were observed during karate practice in those with a high level of karate practice (black belt). However, these results must be interpreted with caution as there was no way to control the multiple factors that might also affect inmate behaviors in a correctional setting. We suggest that karate practice in prison may positively contribute to interactional behaviors.

## Introduction

Practicing karate in a prison environment raises the issue of whether that activity might develop or limit aggressive behaviors and contribute to the rehabilitation process.

The present study was conducted at the Remand Home of Fresnes, France. A remand home (*Male Remand Home* for men, *Female Remand Home* for women) is a detention center for defendants in detention pending trial or for prison inmates whose remaining sentence does not exceed 2 years. Karate is organized there as part of a public service contract between the Val de Marne League of Karate and Associated Disciplines [a decentralized body of the French Federation of Karate and Associated Disciplines (FFKDA)] and the Remand Home of Fresnes. The remand home includes three *Male Remand Home* divisions and the *Female Remand Home*.

Karate was first introduced in a detention setting many years ago, but the FFKDA did not sign an agreement with the Ministry of Justice enabling prison inmates to practice the discipline until 2004. Twelve centers currently offer karate as an activity on a regular basis. The main objectives of the program are described on FFKDA’s (2014) website: to prevent the social rehabilitation of prison inmates through karate. The Ministry of Justice guidebook for physical and sport activities in penitentiary settings states that rehabilitation and reintegration programs involving sports help prison inmates behave in a positive manner and avoid recidivism. Sports also help them deal with physical and psychological issues and generally contribute to improving health and self-image (Ministry of Justice, 2014a, b, c). These elements are emphasized equally and lead us to rethink the issue of rehabilitation (Gras, 2003, 2005; Verdot, 2008).

Here, we use the term “rehabilitation” according to the meaning assigned by Goffman (1973, 1984) as the set of regulatory behaviors with which a prison inmate is required to comply, as a role he/she is willing to play in order to be released from prison and to avoid a repeat offense. Consequently, our question is: because of the specific behaviors that it aims to teach, can karate practice help reach that objective?

### Literature Review on Aggressiveness in Combat Sports

Two articles focus on the fact that aggressiveness can decrease as the number of years of karate practice increases (Nosanchuk, 1981; Lamarre and Nosanchuk, 1999). The first states that “longer training is associated with lower aggressiveness” (Nosanchuk, 1981, p. 435). Therefore, in the light of this work, karate may serve as a tool for improving self-control. The second reports that “a decline in aggressiveness with a training (…) of karate or taekwondo” occurs (Lamarre and Nosanchuk, 1999, p. 992).

These researchers position themselves in favor of the practice of martial arts in general, and karate in particular, as part of a search for self-control and a decrease in aggressiveness. However, we cannot base this study solely on their theoretical framework because it is based essentially on sample groups composed of students and not prison inmates. Other works dealing with these issues introduce an exhaustive collection of conclusions, an “overview of selected studies on martial arts and personality traits,” taken from various studies on martial arts practices, combat sports, and aggressiveness (Vertonghen and Theeboom, 2010, p. 531).

Two major karate studies demonstrate several benefits of this sport practice. First, they found a positive impact on “emotional well-being” in a population of elderly karate practitioners, trained in “tasks of self-defense, partner training, and katas” (Jansen and Dahmen-Zimmer, 2012). Second, other research demonstrates significant benefits in attentional network improvement with a regular practice of martial arts (Johnstone and Mari-Beffa, 2018). Practicing martial arts is acknowledged as a possible tool for self-regulatory behavior, at least in children (Lakes and Hoyt, 2004). Also, new evidence supports karate, especially with long-term practitioners, in the search of mental and physical benefits; karate might be of use in psychosomatic intervention (Vera et al., 2018). We must remain cautious when considering all these studies and previous results, because they are based on a variety of age groups, from the elderly to young children, and results are achieved through long-term practice.

More recently, two researches provided new elements on martial arts and combat sports. First, long-term practice of martial arts reduces anxiety in different sample groups (sex, gender, and level), confirming that the practice of martial arts leads its practitioners to develop emotional stability, the cause of the decrease in anxiety (Fernández et al., 2020). Second, it may also help inmate practitioners improve critical thinking and, similarly, self-control and even mental health (Janelle, 2015). With regard to these elements, Breitschuh et al. (2018) state that club practitioners’ brain gray matter observed through medical imaging “may indicate a reduced need of inhibitory cognitive control because of their improved self-regulation skills.”

Additional researches favor the practice of martial arts for behavioral inhibition and aggressiveness control. Taekwondo, for example, focuses on behavioral control (Lakes et al., 2013). However, it is acknowledged that the longer sports and martial arts are practiced, the better its many skills are learned, included behavioral ones (Witte et al., 2015). Affective stability and mood are also improved more efficiently through sports training and life experiences (Espinoza-Venegas et al., 2015).

On the other hand, it is acknowledged that the practice of sports during adolescence increases verbal aggressiveness and anger, especially if the sport involves contact, and even more if it is a combat sport (Malinauskas et al., 2014). Based on questionnaires, Malinauskas et al. (2014) lean toward the hypothesis that sports may induce aggressiveness. Importantly, aggressive and antisocial behaviors are increased during adolescence and must be studied specifically to determine whether this effect is limited to adolescents or is a persistent behavior (Moffitt, 1993). Indeed, their study does not consider physical aggressiveness or differences between sports practitioners and non-practitioners.

In combat sports, aggressive behavior may be tolerated and might appear equivocal or ambivalent, in terms of what is allowed and what is prohibited (Goldstein, 2011). Ross (2014) acknowledges that authorization of a combat sport by an institution makes it possible to provide education on rules through sports in educational, social, and professional settings. However, at the same time, this authorization leads to institutional recognition of aggressive behavior. The “sport aggressiveness” mentioned by Ross (2014) consists in provoking, intimidating, bullying, marking, communicating, and counter-communicating in a way that is authorized by the rules. These are licit and necessary practices used to enable cooperation with partners and to confront opponents.

From this perspective, it appears necessary to use the classification describing different forms of aggressiveness. Collard (2004) explains that various forms of aggressiveness, which are considered either licit or illicit, are expressed in sports. Licit forms of aggressiveness are understood and permitted by the rules, whereas illicit forms are prohibited.

In the present study, we use a cross-sectional design by observations to assess the forms of licit aggressiveness (permitted and controlled aggressive behaviors included in the rules of the sport) during karate practice. Licit aggressiveness can be observed in *praxic* and *kinesic* motor aggressiveness (Parlebas, 1999). A *praxic* or launched action is a motor activity such as a kick or punch directed toward the opponent, as well as the entire system of counter-communications and tactical situations such as an attacker/defender situation. *Kinesic* aggressiveness or “*kiai*” is a provocation cry. Observation of the bow, which is a sign of respect, can be included in the observation as the opposite of aggressiveness. In the rules of karate practice (FFKDA, 2015), practitioners are required to bow before and after every exercise, and every time they face and leave a training partner/opponent. Bowing is a compulsory courtesy, practiced numerous times in every training session, and not bowing is considered a fault and a very aggressive gesture.

### Literature Review on the Effects of Sports in Prison

At this point, we propose a parallel between the positive results of a meditation and mindfulness program in prison used to combat physical and even mental disorders (Lyons and Dustin Cantrell, 2016) and the practice of the *kata* in karate (a technical form practiced alone).

Motor behaviors performed by practitioners while practicing sport activities—in a systemic and multifactorial approach—enable practitioners to reach objectives such as socialization, integration, insertion, and improvement of health, among others (Bordes et al., 2007). Introducing sports in detention may help prison inmates deal with anxiety and stress (Nelson et al., 2006; Buckaloo et al., 2009; Martos-Garcia et al., 2009), prevent depression (Cashin et al., 2008), adapt and improve their behavior in prison to reduce norm and rule-breaking behaviors (Bushway et al., 2003; Nelson et al., 2006; Meek, 2014; Meek and Lewis, 2014) and decrease aggressiveness, anger, violence, and prison incidents (Wagner et al., 1999; Martos-Garcia et al., 2009). While the practice of sports in prison is a very efficient tool to improve prison inmate health and promote social rehabilitation (DeMaeyer, 2009; Gallant et al., 2015), methods and education programs are necessary to achieve that goal, and the sport should not be practiced solely for the only purpose of physical activity, which could be counterproductive (Martos et al., 2009). By contrast, promoting an activity such as karate might not be recommended in the context of detention, and its effectiveness depends on the inmates’ ability to produce licit aggressiveness (Goldstein, 2011; Malinauskas et al., 2014). Without this ability, karate would not encourage cohabitation. Indeed, the practice of sports in prison may lead to violent and antisocial behaviors (Otto, 2009), especially if management and educational purposes are lacking where rehabilitation is identified as a goal (Coalter, 2007; Meek, 2014). It may also lead to physical injuries in prison inmates (Meek and Lewis, 2012).

Some researchers support the hypothesis that karate training increases aggressiveness in prison inmates (Verdot, 2008; Goldstein, 2011; Malinauskas et al., 2014), whereas others see no differences in aggressive behavior when comparing prison inmates with club practitioners (Vertonghen and Theeboom, 2010). However, a third group of authors suggests that sports in prison offer benefits, including the ability to lower aggressiveness (Gras, 2005; Lakes et al., 2013; Espinoza-Venegas et al., 2015; Gallant et al., 2015; Janelle, 2015; Witte et al., 2015; Lyons and Dustin Cantrell, 2016; Breitschuh et al., 2018). Sports offer practitioners a therapeutic process and may assist them in working on self-control, rehabilitation, and in understanding the prison environment (Gras, 2005; Lyons and Dustin Cantrell, 2016). Gallant et al. (2015, p. 10) state that “sport and recreation programs can improve physical and mental health.” However, no studies have yet been conducted *in situ* examining karate and its relationship with aggressiveness in prison settings.

It is important to report that the records of teaching sessions and staff-led interviews in Remand Homes conducted by one of the authors (Personnel Pénitentiaire, Professeurs de Sports Pénitentiaires et Direction de la Maison d’Arrêt de Fresnes, 2014) show rule-breaking situation observed during karate classes since the sport’s introduction. Also, in addition to the observed results regarding the learning of bows and the control of licit forms of aggressiveness (whether *praxic* or *kinesic*) proposed in Section “Subjects and Methods,”staff-led interviews report that the number of breaches and other rule-breaking behavior in the prison environment by prison inmates practicing karate has been reduced by 90%.

So, is licit aggressiveness of any help in the rehabilitation process? This question leads us to propose a research hypothesis:

Karate practiced in a prison setting is paired with a lower level of aggressiveness in practitioners than karate practiced in a club environment.

## Subjects and Methods

A global educational program is being followed at the Remand Home of Fresnes for karate training (FFKDA, 2014; Ministry of Justice, 2014a, b, c; Personnel Pénitentiaire, Professeurs de Sports Pénitentiaires et Direction de la Maison d’Arrêt de Fresnes, 2014). This program includes prison inmate practice and staff organization to allow it to happen. Behavioral education and the possibility of prison inmate rehabilitation rely on this (Moscoso-Sánchez et al., 2017).

In this study, an observation grid measuring licit aggressiveness was used. We defined licit aggressiveness as the elements of this behavior permitted by karate rules and regulations (FFKDA, 2015), and illicit aggressiveness as breaches and other rule-breaking behavior in the prison environment.

Forms of licit aggressiveness in karate are as follows (Parlebas, 1986; FFKDA Regulations of Grades, 2015):

– The guard’s distances, spaces and amplitudes (i.e., increase and reduction during practice), methods of counter-communication, weapons, targets, the distance of motor confrontation, the individual space of interaction, and contact violence. We call these elements “launched actions.”
– Shouts or sounds (i.e., “*kiais*”) serving a dual purpose: to optimize muscle contraction and to intimidate the opponent.
– Bows, represented by a forward inclination of the trunk between two karatekas. Bows can be linked to any form of licit aggressive intention and are imposed by the federal codes of practice (FFKDA Regulations of Grades, 2015). Bows can be historically traced back to neo-Confucianism, and students bow frequently during a karate class (Draeger, 1974; Didier, 1988; Funakoshi, 1993).

All records were established by the observers according to the determined criteria (Frigout, 2016). We attempted to observe elements related to karate in the framework of its inner logic and the forms of aggressiveness expressed in its practice:

– Launched actions (motor actions in attack, parry-riposte, dodging and counter-attack situations). These actions can be observed in technical and tactical–technical situations.
– *Kiais* or shouts.
– Bows or courtesies expressed throughout practice. These civilities are part of motor and sociomotor practice and belong to the cultural and regulatory frame of reference. Bows are included in the “Regulation of Grades of the Specialized Commission for Dans and Grades and Equivalents” and constitute a genuine social code to be acquired (FFKDA Regulations of Grades, 2015). This regulation, which is specific to karate practice, requires that every practitioner bows before and after exercises. This exercise of civility is incorporated in every motor practice, whether alone, in sparring, or in a group. Although it is taught explicitly during the introductory period of the practice, bowing appears to become self-imposed in experienced practitioners, with no need for any external command.

Table 1 shows the level of aggressiveness, from −2 to 2 (i.e., 0 is an average, based on concrete examples from actual training situations; see Table 1 for examples).

**TABLE 1:** Possible examples of evaluation criteria that can be used to fill the observation grid.

**Aggressiveness in launched actions**	
2 (very aggressive)	Several criteria including distance allowing skin touch, balance, efficient targeting, movement and technical control (see the regulation of grades of the CSDGE) associated with the application of speed and strength (brief and intense contractions).
1 (aggressive)	Several criteria (see above) associated with the application of a physical quality, such as speed or strength.
0 (averagely aggressive)	Several criteria (see above), but with no application of a physical quality, such as speed or strength.
−1 (low aggressiveness)	One criterion (see above), but with no application of a physical quality, such as speed or strength.
−2 (very low aggressiveness)	No criterion (see above) and no application of a physical quality, such as speed or strength. No involvement in the motor exercise.
**Aggressiveness in “*kiais*”**	
2 (very aggressive)	An intense cry and a search for extreme efficiency, showing decisiveness.
1 (aggressive)	A cry and a search for strength and speed.
0 (averagely aggressive)	An audible sound exhalation.
−1 (low aggressiveness)	A low-sound exhalation.
−2 (very low aggressiveness)	No cry, an inaudible or non-existent exhalation. No involvement in the kinesic exercise.
**Aggressiveness in bows**	
2 (very aggressive)	No bow in return to the partner.
1 (aggressive)	Bow performed very quickly while carrying out another task.
0 (averagely aggressive)	Bow performed quickly, glancing at the work target.
−1 (low aggressiveness)	Bow performed slowly, glancing at the work target.
−2 (very low aggressiveness)	Bow performed slowly, glancing at the floor.

**The examples target the forms of praxic and kinesic aggressiveness (launched actions and “kiais”) as well as bows*.*

All observations were made during karate lessons (in prison for prison inmates and at the club for the comparison group). Each of the lessons lasted 1 h 30 min.

The three sports instructors involved in data collection were trained in observation by one of them (the author). They were highly experienced, all holding a black belt and a karate instructor state diploma. The three observers were present during 203 observations, during which two of them were active observers and completed paper grids during karate lessons (Table 1) to measure the level of aggressiveness. After the classes, the data were transferred to an Excel database. Penitentiary staff did not permit video recording. Sixty-two additional observations were made by a single observer present during the lessons. It is important to note that all of the karate classes, both in the club and in the prison, involved a high level of physical energy expenditure. The training sessions were energetic, and the focus included the cultural and technical–tactical parameters of karate (such as *kihon, kumite*, and *kata* exercises). These specific karate exercises were alternated with muscle stretching and strengthening to provide complete physical preparation.

### Participants

Elements of aggressiveness during defined karate training were observed between February 22, 2013, and May 26, 2015, in two distinct study populations in order to compare the measurement of licit aggressive behavior in both of the participant populations: prison inmates and club practitioners.

#### Prison Inmate Population

The observations were made at the Remand Home of Fresnes (three Male and one Female Remand Home divisions) and at the Remand Home of Fleury-Mérogis (for only one practice session with four female prison inmates).

The Male Remand Home of Fresnes houses 2,508 male prison inmates. Of these, 988 practice at least one sporting activity, and 55 (2.1%) prison inmates practice karate (out of all prison inmates in all three divisions). The Female Remand Home houses 75 prison inmates, 45 of whom participate in sports, including 20 (26.6%) who practice karate.

At the Remand Home of Fleury-Mérogis, only one practice session was held with four female inmate karate practitioners who demonstrated the activity to the penitentiary staff and fellow inmates. However, karate was not a regular activity during the observation period.

The prison inmates had been practicing karate for periods of between a few weeks and 2 years and trained between one and three times a week. A total of 61 observations were made for 55 male prison inmates practicing karate, and 16 observations were performed for 16 female prison inmates (including four at the Remand Home of Fleury-Mérogis during a presentation class). One male and one female prison inmate had a black belt. Fifty percent of the male and female inmates practiced 1–2 h of natural bodybuilding per day. The reasons for detention were confidential and not available. Both the male and female inmates had registered to participate in this activity, and all were volunteers. All of the inmates who volunteered to participate in the study were included. No prison inmate, whether male or female, withdrew from the study. However, it is not possible to presume the inmates’ type of motivation, whether intrinsic or extrinsic (Deci and Ryan, 2002).

Information concerning the participants is presented in Table 2.

**TABLE 2:** Characteristics of participants: sex, age, karate level, time of practice.

Gender	*n*	Participant	*n*	Karate level	*n*	Time of practice	*n*
**Descriptive table**							
Female	71	Prison inmates* (age *M* = 30.70; *SD* = 8.81, min = 18, max = 50)	16	White belt	13	<3 years	13
				Colored belt	2	<3 years	1
						>3 years	1
				Black belt	1	>3 years	1
		Club practitioners (age *M* = 23.35; *SD* = 7.42, min = 18, max = 43)	55	White belt	9	<3 years	9
				Colored belt	37	<3 years	20
						>3 years	17
				Black belt	9	>3 years	9
Male	194	Prison inmates** (age *M* = 33.58; *SD* = 9.22, min = 25, max = 65)	61	White belt	59	<3 years	59
				Colored belt	1	<3 years	1
				Black belt	1	>3 years	1
		Club practitioners*** (age *M* = 25.29; *SD* = 8.67, min = 18, max = 61)	133	White belt	10	<3 years	10
				Colored belt	108	<3 years	49
						>3 years	59
				Black belt	15	>3 years	15

*One convict passed the national karate certificate of sport educator (DEJEPS) during the detention. **Six convicts practiced combat sport during the detention. One convict was a wrestler (Greco-roman) and performed the Olympic Games in Beijing (2008). One convict was European champion of full-contact. ***Two practitioners practiced mixed martial arts. One practitioner practiced taekwondo and savate-French boxing.

#### Comparison Group

A karate club formed the comparison group. The practitioners had been practicing for between a few weeks and 20 years, and 12 of them (three females and nine males) had acquired a “*dan*” rank (grades within the black belt, from first to fifth *dan*). The practitioners trained two or three times a week. A total of 188 observations were performed for 117 practitioners: 133 observations for 80 males, and 55 for 37 females, all of whom were members of the FFKDA. Twenty percent of the male and female groups performed two or three sessions of natural bodybuilding or fitness practice per week.

Information concerning the participants is presented in Table 2.

### Indicators of Licit Aggressiveness

We used observation grids based on Dugas (2006) for observations in basketball, identifying licit aggressiveness on a scale from −2 to 2, 0 being the average (Frigout, 2016).

The observation grid of licit aggressive practices (praxic and kinesic) was completed on paper during observation of the discriminating elements (level of licit aggressiveness in launched actions, kiais, and bows). The recorded data included the number of educational situations including actions that were initiated and performed by the practitioner, the number of educational situations involving *kiais*, and the number of educational situations involving bows. For each of these items, an observation record identified the level of aggressiveness using the following precise scale from −2 to 2: −2: very low aggressiveness; −1: low aggressiveness; 0: average aggressiveness; 1: aggressive behavior; 2: very aggressive behavior.

### Statistical Analysis

A total of 203 sessions (64 in prison and 139 in a club setting) were analyzed, representing 76.6% of all (265) observations, representing more than the minimum acceptable value of 10% (Tabachnick and Fidell, 2010). The observers completed their grids separately during the classes and rated them after classes in an Excel database for comparison. Two of the three possible observers during the 203 observations were active. Cohen κ was applied. The interrater reliability for the “aggressiveness of launched actions,” “aggressiveness of *kiais*,” and “aggressiveness of bows” was 1.00. This perfect (100%) reliability can be explained by the accuracy of the criteria presented in Table 1.

This research focuses on the evaluation of aggressive behavior, comparing prison inmates and club practitioners during karate practice. The normality of the variables (level of aggressiveness in launched actions, *kiais*, and bows) was tested using the Kolmogorov–Smirnov test. The data showed a non-normal distribution. Comparisons by group (prison inmates and club practitioners), gender (female and male), and belt (level) according to “aggressiveness of launched actions,” “aggressiveness of *kiais*,” and “aggressiveness of bows” (three forms of licit aggressiveness in karate) were performed using the non-parametric Mann–Whitney *U* test for independent samples. Data were analyzed using SPSS for Mac (V.23; SPSS Inc., Chicago, IL, United States). Statistical inference was performed at the level of significance of *p* < 0.05.

Significant main effects of each variable were followed up using Bonferroni-corrected analysis. The level of significance after Bonferroni correction is set at *p* < 0.003. Effect size (η^2^) values of 0.2, 0.5, and 0.8 were considered to represent small, medium, and large differences, respectively (Cohen, 1988).

### Ethical Considerations

No observations were made in a competitive situation (i.e., the prison inmates were not permitted to leave the prison). All subjects gave informed written consent as required by the Declaration of Helsinki. The experiment was approved by the local ethics committee of Université Paris-Saclay (affiliations: EA 4532; CIAMS, Université Paris-Sud, Université Paris-Saclay, Orsay, France; CIAMS, Université d’Orléans, Orléans, France).

Under the agreement with the Ministry of Justice for the practice of karate in detention, the FFKDA has no access to prison inmates’ files. Federal members and sport educators, who participate in the observations, are not permitted to attend the commission for the application of penalties (for ethical and deontological reasons). Certain prison inmates (who have committed numerous infractions) are not permitted to participate in sport activities.

## Results

Of a total of 265 observations, 77 were performed with prison inmates and 188 with club practitioners outside prison (comparison group).

### Results Concerning the Aggressiveness of Launched Actions, Kiais, and Bows for Female and Male Participants

Table 3 presents the results by gender concerning the level of aggressiveness of launched actions, *kiais*, and bows.

**TABLE 3:** Mean, standard deviation, and significance of Mann–Whitney test concerning the level of aggressiveness in launched actions, “*kiaïs*” and bows for male and female practitioners separately, by groups.

		Participants	*n*	Mean	*SD*	*p*	η^2^
Female	Aggressiveness launched actions	Club practitioners	55	1.60	0.63	<0.001	0.40
		Prison inmates	16	–0.19	1.17		
	Aggressiveness *kiaïs*	Club practitioners	55	1.44	0.71	<0.001	0.43
		Prison inmates	16	–0.31	0.87		
	Aggressiveness bows	Club practitioners	55	–2.00	0.00	<0.001	0.69
		Prison inmates	16	–0.94	0.85		
Male	Aggressiveness launched actions	Club practitioners	133	1.75	0.56	<0.001	0.16
		Prison inmates	61	1.13	1.01		
	Aggressiveness *kiaïs*	Club practitioners	133	1.41	0.85	<0.001	0.19
		Prison inmates	61	0.46	1.07		
	Aggressiveness bows	Club practitioners	133	–1.99	0.87	<0.001	0.57
		Prison inmates	61	–0.77	1.09		

**Significance level set at p < 0.003 after Bonferroni adjustment*.*

The level of aggressiveness during launched actions, *kiais*, and bows differed significantly between the groups for both male and female practitioners (Table 3). There was a significant difference in the results for “prison inmates” and “club practitioners” for launched actions, *kiais*, and bows (*p* < 0.001; Table 3). The results showed that there were significant differences in the level of aggressiveness during karate practice for both male and female practitioners when comparing prison inmates and club practitioners. While the level of aggressiveness was lower during launched actions and *kiais*, it was higher during bows for prison inmates compared to club practitioners.

There was a significant difference in the aggressiveness level in launched actions (−0.19 mean, Table 3) and *kiais* (−0.31 mean, Table 3) between the female inmates group and the three others groups (female in club, male prison inmates, and male in club), with the aggressiveness level in launched actions of the female inmates group being lower than the other groups.

### Results Concerning the Aggressiveness of Launched Actions, Kiais, and Bows in Relation to the Level of Practice (Belts)

Table 4 presents the results of the observations made in this study concerning the level of aggressiveness of launched actions, kiais, and bows, considering the participants’ level of practice. In karate, beginners wear a white belt (“white belt” in the table), advanced practitioners wear colored belts (“*kyu*”), and experts wear a black belt (“*dan*”). Three years is the minimum legal period of full-time practice to obtain a “*dan”* rank in France. This 3-year practice time limit is the minimum to take the black belt examination (first *dan*), according to the CSDGE rules established by the French Ministry of Sports (FFKDA Regulations of Grades, 2015). Three years is also considered long-term practice in studies measuring positive psychobiological effects (Vera et al., 2018).

**TABLE 4:** Mean, standard deviation and significance of Mann–Whitney test concerning the aggressiveness of launched actions, “*kiaïs*” and bows considering the level (belts) of the participants.

		Participants	*n*	Mean	*SD*	*p*	η^2^
White belt	Aggressiveness launched actions	Club practitioners	19	0.95	0.78	0.930	
		Prison inmates	72	0.82	1.19		
	Aggressiveness *kiaïs*	Club practitioners	19	0.84	0.69	0.043	
		Prison inmates	72	0.29	1.08		
	Aggressiveness bows	Club practitioners	19	–2.00	0.00	< 0.001	0.29
		Prison inmates	72	–0.75	1.05		
Colored belt *(kyu)*	Aggressiveness launched actions	Club practitioners	145	1.79	0.47	0.001	0.04
		Prison inmates	3	1.00	0.00		
	Aggressiveness *kiaïs*	Club practitioners	145	1.44	0.82	0.011	
		Prison inmates	3	0.00	1.00		
	Aggressiveness bows	Club practitioners	145	–1.99	0.08	< 0.001	0.44
		Prison inmates	3	–1.33	0.58		
Black belt *(dan)*	Aggressiveness launched actions	Club practitioners	24	1.79	0.59	0.812	
		Prison inmates	2	2.00	0.00		
	Aggressiveness *kiaïs*	Club practitioners	24	1.79	0.51	0.394	
		Prison inmates	2	1.00	1.41		
	Aggressiveness bows	Club practitioners	24	–2.00	0.00	< 1.00	
		Prison inmates	2	–2.00	0.00		

**Significance level set at p < 0.003 after Bonferroni adjustment.**

While the averages for “prison inmates” and “club practitioners” showed very different results for launched actions, *kiais*, and bows for “white belt” participants (*p* = 0.062 for launched actions, *p* = 0.043 for *kiais*, *p* < 0.001 for bows; Table 4), “colored belt” participants had averages with highly significant differences (*p* < 0.001 for launched actions and *p* < 0.001 for bows; Table 4). There was no difference in the averages for “prison inmate” and “club practitioner” black belts (*p* = 0.054 for launched actions, *p* = 0.026 for *kiais*, *p* = 0.067 for bows; Table 4), although the samples were very small and should be considered possible limits.

There were significantly more “*kyu*” and “*black belt*” club practitioners than “*kyu*” and “*black belt*” prison inmates. There were 108 + 15 (123) with a colored belt and 10 beginners in club practitioners, and 1 + 1 (2) with a colored belt and 59 beginners in prison inmates in the male population. Ninety-seven percent of male and 81% of female prison inmates were beginners, compared to only 7.5% of male and 16.4% of female beginner club practitioners. This is a significant difference in the level of knowledge of karate that should also be discussed.

## Discussion

### Differences in the Aggressiveness Level Between Groups

There is a sign difference in the aggressiveness level in both female and male practitioner groups during launched actions, *kiais* (lower for prison inmates), and bows (higher for prison inmates).

In club karate practice, strong *praxic* and *kinesic* aggressiveness was associated with a high level of civility, whereas in detention, we observed less *praxic* and *kinesic* aggressiveness but also less respect for bows (courtesies). Despite the lack of respect for bows in prison, karate appeared to have reached the goals set by the penitentiary administration.

It has been proved that, even in the prison environment, aggressive behavior can be counterbalanced with different physical activities (Kerekes et al., 2017).

The practice of traditional martial arts may help address or reduce aggressiveness and violence or even develop psychological well-being (Nosanchuk, 1981; Lamarre and Nosanchuk, 1999; Vertonghen and Theeboom, 2010). Our results strengthen previous finding that karate practice in prison might help in controlling aggressiveness (Breitschuh et al., 2018) and developing self-regulation behavior skills (Lakes and Hoyt, 2004; Lakes et al., 2013) and might improve mental and physical health in general (Vera et al., 2018). We recall that the practice of karate in prison can help develop emotional skills (Fernández et al., 2020) and also appears to help improve critical thinking, self-control, and mental health in prison inmates (Janelle, 2015).

The higher aggressiveness level revealed in the prison inmates during bows could be interpreted as reflecting a lower level of trust and respect (that is reversed over time for black belts). Both the lower aggressiveness level in beginner prison inmates during launched actions and *kiais* and the higher level of aggressiveness during bows indicate a greater degree of insecurity, mistrust, anxiety, and fear. Each of these could be associated with the situation (detention in prison) and their specific personality profile [described with lower degree of cooperativeness, and self-directedness, paired with greater level of harm avoidance (Falk et al., 2017)].

Aggressive antisocial behavior can be alleviated through increased self-governance therapies including the practice of sport (Kerekes et al., 2019).

Although some authors, such as Ross (2014), consider there to be a socially permitted and acknowledged athletic form of aggressiveness, we do not find that categorization to be defined clearly enough. The form of aggressiveness that she examines and observes through the use of personality questionnaires can relate as much to socially reprehensible behaviors as to the form of aggressiveness required in sports.

In our study, we intend to question the inner logic specific to the karate activity within which aggressiveness, although controlled, is also aroused (Collard, 2004). In the light of these observations, we believe that karate can induce an acceptance of collectively shared rules, not through an attenuation of aggressive behaviors but by calling upon practitioners to integrate the logic of that activity and achieve the associated technique. The results seem to confirm this, because black belt practitioners in both groups show no differences in their engagement in the aggressiveness of karate practice, whether in launched actions, *kiais*, or bows (although, we repeat, these specific samples are small, and the null size effect should be considered an important limitation). While the significance of the aggressiveness level results for males and females also appears to indicate lessened aggressiveness in the context of detention when compared to a club environment, we must not conclude too quickly in favor of the practice of karate. During the study, the prison inmates were being observed and were aware of that fact. They are required to register for sports activities, await a positive response, and can be banned from the sporting activity in case of misbehavior. Also, they distrust and fear each other in the framework of their prison environment. A system is also in place allowing an individual to be expelled from a given activity should they display illicit aggressiveness, violence, or rule-breaking behavior. The distrust of prison inmates can be analyzed through the observation of aggressiveness in bows (they bow quickly, or glance the training partner), which is significantly different from the club practitioners. These hypotheses will have to be verified through further research.

This study tends to favor conducting and maintaining karate practice as part of the detention process. No incivility was observed during the karate activity by either the caseworkers or the penitentiary staff between 1993 and 2015, and no breach was committed by the prison inmates during their practice sessions. Regarding the improvement of behaviors and the acceptance of community rules by the prison inmates, penitentiary staff noted a large decrease in incivility and other breaches committed by practitioners from the moment they entered the karate practice (Personnel Pénitentiaire, Professeurs de Sports Pénitentiaires et Direction de la Maison d’Arrêt de Fresnes, 2014). This important piece of information was provided by the penitentiary staff to the first author, who was also one of the observers. Furthermore, no one had ever been expelled from karate class (no case of illicit aggressiveness, violence, or rule-breaking behavior).

Prison inmates are invited to practice karate at the Remand Home of Fresnes for their interpersonal well-being, and we observed that participants implemented aggressiveness strategies (whether praxic or kinesic) to a lesser degree than do practitioners in clubs. This finding, combined with lower respect for civility during practice (bows), does not necessarily prove, as may have already been demonstrated in other research, that even combat sports can have a place in detention, serving to (re)habilitate individuals and improve cohabitation (Gras, 2003, 2005).

We confirm and retain our hypothesis proposed at the beginning of this article: there was less aggressiveness in the motor behaviors of prison inmates than in those of club practitioners.

### The Decrease in Aggressiveness Level Differences Between the Groups With Time of Practice and Experience, and the Behavioral Control of Black Belt Practitioners (Licit Aggressiveness), in Both the Prison Environment and Clubs

Observation of beginners showed that licit aggressiveness was lower in prison inmates. This behavior may be explained by a high level of psychological distress (including anxiety, phobic anxiety, paranoid ideation, hostility, etc.) (Edwards and Potter, 2004). Also, those with a higher level of knowledge in martial arts were able to express licit aggressiveness even in prison, suggesting that with time, karate practice increases emotional and behavioral control (coupled with a higher *praxic* and *kinesic* aggressiveness level and greater respect for bowing). Previous studies have shown that sport activities in prison have the capacity to increase the maturity of prison inmates (Kerekes et al., 2019), which can be measured on increased impulse and behavioral control, decreased aggressive and antisocial behaviors (Kerekes et al., 2017), and even on decreased level of psychological distress (Kerekes et al., 2018; Sfendla et al., 2018).

As previous studies revealed that affective stability and mood are better controlled and dealt with through longer training and long-term programs (Espinoza-Venegas et al., 2015; Witte et al., 2015), we can assess the significance of our results concerning the time of practice above 3 years in the black belt participants. These results also highlighted a possible limitation of our study: direct observation needs to be performed over an extended period of time to determine its efficiency.

We can add that the observations in the present work are not inconsistent with the results of Gras (2003, 2005), who demonstrated how physical and sport activities are driving forces in reshaping identity in detention, both individually and socially.

These results concerning “emotional well-being” and “attentional network” improvements are directly correlated with karate and martial arts practice (Jansen and Dahmen-Zimmer, 2012; Johnstone and Mari-Beffa, 2018). These conclusions favor karate and martial arts practice as offering several benefits, again both individual and social (Gras, 2003, 2005). However, concerning mental and personal improvements through karate practice in prison, we cannot establish any common points between mindfulness programs in prison (through *kata* practice, for example) to fight physical and even mental disorders (Lyons and Dustin Cantrell, 2016), for this was not the purpose of this study. However, while several benefits such as channeling aggressiveness into a licit form and even reducing it are targeted by this study, the results presented cannot fully conclude in favor of this hypothesis.

The practice of sports in prison can provide effective results on the possible adjustment, treatment and regulation of anxiety and stress (Nelson et al., 2006; Buckaloo et al., 2009; Martos-Garcia et al., 2009), depression (Cashin et al., 2008), behavior adaptation (Bushway et al., 2003; Nelson et al., 2006; Meek, 2014; Meek and Lewis, 2014), aggressiveness, anger, violence control, and a reduction in prison incidents (Wagner et al., 1999; Martos-Garcia et al., 2009). The present study’s results converge with those mentioned above, although we must remain cautious because some of the samples of this study are small (prison inmate colored belts, black belts), the size effects of launched actions and *kiais* are very small for male participants and null to very small for launched actions for all types of belts, and the bow size effect is null for black belts.

Sports and karate in prison help promote social rehabilitation as previously corroborated by DeMaeyer (2009), as methods and global educational karate programs are pursued (Martos et al., 2009).

As we complete this study, we believe it necessary to acknowledge that karate uses rather than reduces licit aggressiveness in practitioners, while creating cohabitation. By that we mean that karate may allow prison inmates to become closer as they share time together during detention. During that time, they may share space, training, and rules and can cooperate or oppose each other. For prison inmates, that time is spent socializing. We recall that in this study the results show no significant differences between practitioners and prison inmates for both males and females, although a significant difference exists between female prison inmates and the three other groups in terms of their aggressiveness in launched actions (−0.19 mean) and *kiais* (−0.31 mean), which are lower than those of the other groups. We suppose that this difference might be due to the inexperience of this sample, for only one female prison inmate had practiced karate before entering prison (more than 3 years of experience acquired partly outside prison), while in the male prison inmates sample, eight had previous combat sport or martial arts experience, which might influence their behavior during the activity. We also note that gender differences are not significant for black belt participants, even in the case of bow aggressiveness (although the samples are small in those particular cases). Self-regulation skills research by Breitschuh et al. (2018) in their study on martial artists’ aggressiveness and gray matter concentration should be a complementary tool to be applied in prison (and also on a female sample), which may offer new evidence and conclusions. But here again, a long-term study seems necessary to complete our findings and direct observations.

The lessened aggressiveness observed among beginner prison inmates may reflect a form of heightened caution exercised among themselves, as presented above. We might also observe a link with cohabitation in that regard: cohabitation may be assimilated with respect for others, although this statement is counterbalanced by less respect for bows.

With the black belt practitioners, we clearly observed a tendency toward increased self-regulation and control: karate enabled them to practice a high level of praxic and *kinesic* aggressiveness, which can be considered as areas of self and collective expression, and lead them to practice bows with greater respect, reflecting more self-confidence and trust in other prison inmates during karate sessions.

Confirming the conclusions of Moscoso-Sánchez et al. (2017) on possible results with a full educational process of rehabilitation in prison, including staff and inmates, these observations can also be seen as evidence of the euphemization and ritualization of violence in sports and an attempt to demonstrate that modern sport practice contributes to “the learning of control and self-control of urges,” while being a “tolerated area to unleash emotions” (Bodin and Debarbieux, 2001, p. 13). Research specifically applied in an empirical study on boxing and aikido (Sánchez Garcia, 2013) also demonstrates this. The multiple causes of that self-regulation or control in prison are merely hypothetical at this stage: are they related to pressure applied by prison inmates among themselves and the fear of acting out or is pressure applied by detention itself, etc.? Also, further research could establish a possible difference in the number of years some practitioners have been involved in karate and its correlation with the self-regulation of aggressiveness.

First, sports offered in prison are not homogenous, and all sports are not accessible. In other words, a prison inmate who wants to try karate may not necessarily be able to do so in the Remand Home in which he/she is incarcerated because the sport has not been set up, because the waiting list is too long or because insufficient means have been invested by the center and the FFKDA, which may not facilitate his/her registration. According the Guide of the Penitentiary Administration of the Ministry of Justice (Ministry of Justice—Guide, 2014; Ministry of Justice—Key Figures, 2014), 90,982 people are currently in detention, but only 56,992 spaces are available for participation in sports in the 190 detention centers in France. In addition, only 300 sports instructors are responsible for developing these practices in partnership with 12 federations having signed agreements with the Ministry of Justice. Considering these gaps and disparities across the country as well as the activities available, we cannot expect sports in general, and karate in particular, to single-handedly offer a solution to managing aggressiveness and possible rehabilitation.

After having conceptualized the concept of cohabitation, we suggest that karate in prison contributes to interactional behaviors, which help to develop the social cohesion sought by the Ministry of Urban Affairs, Youth and Sports by increasing its accessibility to larger target groups of the public (Ferréol, 2003; Zanna, 2011). However, although the acceptance of rules applies in the practice of karate and life in detention, we cannot claim that the acquired knowledge will be transferred after their release and lead to rehabilitation, which is seen by Goffman (1984) and Gras (2005) as a long-lasting transformation outside penitentiary centers.

## Strengths and Limitations

The present study is based on direct observation, offering results on licit forms of aggressiveness practiced in karate during classes, comparing the prison and club environments. In both female and male participants, prison inmates showed less aggressive behavior than did club practitioners in launched actions and *kiais* and more aggressiveness with bows. While beginners (white belts) display less civil behavior, prison inmates seem to evolve in their practice with experience and acquisition of the black belt (1st *dan* and beyond), requiring commitment in launched actions, *kiais*, and bows. In line with these results, prison inmate practitioners are reported to be more respectful of detention rules, with very significant decreases in incivilities, aggressiveness, and violent behavior in prison. However, several limitations appeared because of the small size of some samples in this study, such as experienced black belt prison inmates in comparison to club practitioners. The significant number of beginners among the prison inmates and some of the effect sizes discussed previously are to be considered as limitations for the study of the results.

While direct observation of licit and sporting aggressiveness in prison shows significant results, it also requires further study, using long-term and complete observations with self-reporting to measure the effects of this practice and possible multiple causes of its consequences on aggressive behavior. Further investigation using PAMA (Kerekes et al., 2018) can be used to measure changes in aggressive behavior in the prison population. Introducing the method developed by Breitschuh et al. (2018) to observe the aggressiveness of martial artists through gray matter concentration in prison could help confirm the results of this study.

We also recall a possible limitation of this study: 62 direct observations were made by just one observer, with no possibility for interrater observation reliability, which may represent a bias and a limitation to the results.

## Conclusion

Karate is a suitable and functional form of physical activity for prison inmates. The licit aggressiveness level observed during practice showed two tendencies: beginners expressed a lower level of *praxic* (launched actions) and *kinesic* (*kiais*) aggressiveness than did club practitioners and less respect for bows (courtesies), whereas no differences were observed in experienced karatekas (black belt) between the level of aggressiveness and bows in the two groups. Karate was found to impact emotional and behavioral control (self-regulation and self-confidence). It must be practiced for a long time to allow those effects and to decrease illicit aggressiveness and antisocial behavior. While this educational program is offered at the Remand Home of Fresnes, it should be verified whether this situation is found in all French prisons in which karate is practiced. The complex approach of our research topic will have to be addressed and dealt with in depth in the framework of a more systemic perspective.

## Data Availability Statement

The raw data supporting the conclusions of this article will be made available by the authors, without undue reservation, to any qualified researcher.

## Ethics Statement

The studies involving human participants were reviewed and approved by the local ethic committee of the Université Paris-Saclay (affiliations: EA 4532; CIAMS, Université Paris-Sud., Université Paris-Saclay, 91405 Orsay, France; CIAMS, Université d’Orléans, 45067 Orléans, France). The patients/participants provided their written informed consent to participate in this study.

## Author Contributions

JF designed the study, collected, analyzed, and interpreted the data. OD completed the statistical analysis. JF, OD, and AD drafted and revised the manuscript, prepared the tables, and gave final approval. All authors contributed to the article and approved the submitted version.

## Conflict of Interest

The authors declare that the research was conducted in the absence of any commercial or financial relationships that could be construed as a potential conflict of interest.
